# The Effects of Air Pollution on the Brain: a Review of Studies Interfacing Environmental Epidemiology and Neuroimaging

**DOI:** 10.1007/s40572-018-0209-9

**Published:** 2018-07-14

**Authors:** Paula de Prado Bert, Elisabet Mae Henderson Mercader, Jesus Pujol, Jordi Sunyer, Marion Mortamais

**Affiliations:** 1 Pompeu Fabra University, Barcelona, Catalonia Spain; 2 MRI Research Unit, Department of Radiology, Hospital del Mar, Barcelona, Spain; 3 Centro Investigación Biomédica en Red de Salud Mental, CIBERSAM G21, Barcelona, Spain; 4 ISGLOBAL, Centre for Research in Environmental Epidemiology (CREAL), Barcelona, Spain; 5 Consortium for Biomedical Research in Epidemiology and Public Health (CIBERESP), Madrid, Spain; 6 Institut Hospital del Mar d’Investigacions Mèdiques–Parc de Salut Mar, Barcelona, Catalonia Spain

**Keywords:** Air pollution, Brain, Neuroimaging, Epidemiological studies, Cognition

## Abstract

**Purpose of Review:**

An emerging body of evidence has raised concern regarding the potentially harmful effects of inhaled pollutants on the central nervous system during the last decade. In the general population, traffic-related air pollution (TRAP) exposure has been associated with adverse effects on cognitive, behavior, and psychomotor development in children, and with cognitive decline and higher risk of dementia in the elderly. Recently, studies have interfaced environmental epidemiology with magnetic resonance imaging to investigate in vivo the effects of TRAP on the human brain. The aim of this systematic review was to describe and synthesize the findings from these studies. The bibliographic search was carried out in PubMed with ad hoc keywords.

**Recent Findings:**

The selected studies revealed that cerebral white matter, cortical gray matter, and basal ganglia might be the targets of TRAP. The detected brain damages could be involved in cognition changes.

**Summary:**

The effect of TRAP on cognition appears to be biologically plausible. Interfacing environmental epidemiology and neuroimaging is an emerging field with room for improvement. Future studies, together with inputs from experimental findings, should provide more relevant and detailed knowledge about the nature of the relationship between TRAP exposure and cognitive, behavior, and psychomotor disorders observed in the general population.

## Introduction

Air pollution is a major environmental health problem that has been already associated with cardiovascular morbidity and mortality [1]. Common sources of outdoor air pollution are combustion of fossil fuels and industrial and agricultural processes. Air pollutants that are a major public health concern include particulate matter (PM) (e.g., organic and elemental carbon [EC], metals and polycyclic aromatic hydrocarbons [PAHs]), carbon monoxide (CO), ozone (O_3_), lead, nitrogen dioxide (NO_2_), and sulfur dioxide (SO_2_) (https://www.epa.gov/criteria-air-pollutants).

An emerging body of evidence has raised concern regarding the potentially harmful effects of inhaled pollutants during the last decade on the central nervous system (CNS). Traffic-related air pollution (TRAP) exposure has been associated with adverse effects on mental development and on behavioral functions such as attention, reduced global IQ, a decrease in memory and academic performance, and higher prevalence of Attention Deficit Hyperactivity Disorder and Autism Spectrum Disorder [2•, 3]. Findings from epidemiological studies also provide support for a relation of TRAP exposure to cognitive decline and dementia in the elderly [4–6].

Animal studies have shown that inflammation and oxidative stress, identified as common and basic mechanisms through which air pollution causes damages [7], may also affect the CNS by inducing neuronal death or synaptic toxicity [8–10]. Increasing levels of circulating cytokines due to systemic inflammation may indeed have a peripheral impact on the brain, and/or air pollutants might reach the brain after crossing the blood brain barrier or more directly via the olfactory bulb, and may themselves be proinflammatory [11••, 12].

Damage caused to the CNS has been investigated in experimental conditions with animals via acute exposure to high levels of pollutants [8, 9, 13–15]. Generally, humans suffer a chronic exposure to TRAP at lower levels than those used in the experimental studies [16–20] . The changes occurring in the human brain under these real conditions still have to be precisely defined to provide biological plausibility for the associations between TRAP and adverse effects on cognition reported in epidemiological studies. Magnetic resonance imaging (MRI) is a powerful tool allowing in vivo investigation of brain structure and functioning. Anatomical MRI is used to assess morphological features of the brain such as the whole-brain volume, the volume of specific regions of the brain, and cortical thickness [21]. On the other hand, diffusion tensor imaging (DTI) provides data on white matter integrity and fiber connectivity between brain structures [22]. Functional MRI (fMRI) provides indirect measures of neuronal activity and magnetic resonance spectrometry (MRS) allows in vivo investigation of a number of cerebral metabolites.

Recent MRI applications in environmental health studies have already given promising clues to understanding the relationships between behavioral and cognitive disorders and exposure to lead, pesticides, or tobacco smoke [23••]. The present study systematically reviewed existing literature about interfacing neuroimaging and epidemiology to evaluate the impact of air pollution on the brain.

## Methods

The systemic search of studies was carried out in the PubMed search engine (U.S. National Library of Medicine) until May 2017 using the following keywords: (neuroimaging OR “brain imaging” OR MRI OR fMRI OR “magnetic resonance imaging” OR “functional magnetic resonance imaging” OR “functional MRI” OR “white matter”) AND (“air pollution” OR particulates OR PM2.5 OR PM10 OR “PM coarse” OR diesel OR NO_2_ OR ozone OR “air pollutants”). Identification and first screening of the articles was performed using the information available in the title and the abstract. Doubts regarding the inclusion or exclusion of studies were resolved by discussion between the authors.

## Results

**Fig. 1:**
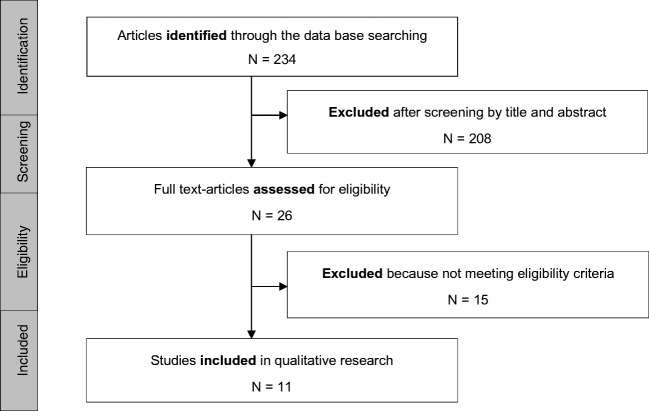
Selection process of the articles

A total of 234 articles were identified in PubMed in our initial search. Twenty-six of them were selected as potentially eligible based on the title and abstract of which 3 articles were excluded because they were reviews, 11 because they were occupational studies, and another one because there was no cerebral neuroimaging data. Finally, 11 articles met the eligibility criteria and were chosen for a full-text evaluation (Fig. 1).

### Study Characteristics

Among the 11 selected studies (Table 1), six evaluated the impact of urban air pollution on children’s brains. Two comparative studies, carried out within small samples of healthy children, compared children with lifetime residency in Mexico City with those from a low-polluted city [24, 25]. A birth cohort, conducted in New York City in a minority population with high poverty and high levels of pollution, used personal air monitoring of the mother over a 48-h period in the third trimester of pregnancy to investigate the effects of prenatal exposure to polycyclic aromatic hydrocarbons (PAHs) on brain structures in 40 children [19]. Finally, the BREATHE project (Barcelona, Spain) explored whether high levels of TRAP in the school environment using a combined measurement of EC and NO_2_ [26], airborne copper [20], or PAHs [18] could be related to disruptions in the brain maturation of 263 children from the general population.

**Table 1:** Air pollution exposure and MRI-detected brain changes in humans and rats, ordered by year and author

Author/year	Population	Study design	Air pollution exposure assessment	Neuroimaging data		Findings
	Sample size age (at MRI) cohort			MRI acquisition	Data processing/outcomes	
*In children*						
[24]	*n* = 36	P	Lifetime residency in high-polluted area (Mexico City) for 23 children versus lifetime residency in low-polluted area (Polotitlán) for 13 children.	MRI 1.5 T	Lesions assessment	Prefrontal WMH in 56.5% of the Mexico City children (compared with 7.6% in Polotitlán children).
	7–18 years			T2 and FLAIR		
					WMH	
						WMH were persistent over time in 3 children followed up during 11 months.
						Similar WMH found in 4 of 7 young Mexico City dogs scanned in this study.
[25]	*n* = 30	P	Lifetime residency in high-polluted area (Mexico City) for 20 children (10 with WMH, 10 without), versus lifetime residency in low-polluted area (Polotitlán) for 10 children.	MRI 1.5 T	Lesions assessment	Children from Polotitlán had significantly more WM volume in temporal and parietal areas than children from Mexico City.
	7–8 years			T2 and FLAIR		
					WMH	
					ROI-based analysis/volumetric measurements	HV, BG volumes, and amygdala were not significantly different across the three groups.
					WM, GM, CSF volumes in brain’s lobes, HV, BG, and amygdala volumes.	
[19]	*n* = 40	L	PAHs	MRI 3 T	Whole-brain voxel-based analysis	No correlations between PAHs and measures of cortical thickness.
	7–9 years		1) Prenatal period (personal air monitoring of the mothers over a 48-h period in the 3rd trimester of pregnancy)	T1-weighted images		
	Birth cohort, NYC					
						Powerful dose-response relationship between prenatal PAH exposure and subsequent reductions of the WM surface (entire surface of left hemisphere).
					Surface morphological measures of GM and WM (index of local volumes).	
						Postnatal exposure correlated significantly with WM measures in dorsal prefrontal regions bilaterally (independently of prenatal exposure).
			2) Postnatal period (Urinary PAH Metabolites in children)			
						*Adjustment for age and sex*
[26]	*n* = 263	C	Weighted annual average of EC and NO2 (marker of road traffic).	MRI 1.5 T	Whole-brain voxel-based analysis	No evident effect on brain anatomy, structure, or membrane metabolism.
	8–12 years			T1-weighted 3D sequence		
	BREATHE study				GM and WM volumes and concentrations, FA, and fMRI signal during task.	However, traffic-related air pollution was related to functional brain changes:
				DTI		
				MRS		
						*1)Resting state fMRI*: Lower functional connectivity between regions distant in space of the DMN (=lower integration), higher functional connectivity between regions close in space (=lower segregation).
				fMRI		
					Whole cortex vertex-based analysis	
			Real measurement of pollutants at the children’s school.	-Resting state		
					Cortical thickness	
				-Task (ABABABAB block design alternating 4 30-s periods of rest with 4 30-s periods of visual–auditory stimulation)	ROI-based analysis	
						*2) Passive fMRI task*: Lower deactivation in the supplemental motor area and somatosensory cortex.
					fMRI signal at resting state in 4 functional connectivity maps (Seed regions: MFC, PCC, DFC, and SMA)	
						*Adjustment for age, sex, academic achievement, difficulties scores, obesity, parental education, home and school vulnerability index, and distance, from home to school and public/nonpublic school category*
[20]			Airborne copper	MRI 1.5 T	Whole-brain voxel-based analysis	Higher copper exposure is associated with
				T1-weighted 3D sequence		
						1) Altered structure of caudate nucleus, 2) delayed maturation of the WM pathways in the caudate nucleus region, and 3) reduction of functional connectivity between the caudate nucleus and the frontal cortex.
			Annual level. Real measurement of pollutants at the primary school.		GM and WM volumes and concentrations, FA	
				DTI		
				MRS	Whole cortex vertex-based analysis	
				fMRI (Resting state)		
						*Adjustment for age, sex, academic achievement, academic difficulties score, obesity, parental education, home and school vulnerability index, and public/nonpublic school category.*
					Cortical thickness	
					ROI-based analysis	
					fMRI signal at resting state in functional connectivity maps (Seed regions: MFC and caudate nucleus)	
[18]	*n* = 242	C	PAHs	MRI 1.5 T	ROI-based analysis/volumetric measurements	Higher exposures to PAHs and BAP were associated with a reduction in the caudate nucleus size. TBV, putamen, and globus pallidum volumes did not differ by PAHs exposure.
	8–12 years		Annual level. Real measurement of pollutants at the primary school.	T1-weighted 3D sequence		
	BREATHE Study					
					TBV and BG volume (putamen, caudate nucleus, and globus pallidus volumes)	
						*Adjustment for age at MRI, sex, intracranial volume, maternal education, and residential neighborhood socioeconomic status*
*In elderly*						
[17]	*n* = 1403 women	P	PM _2.5_	MRI 1.5 T	ROI-based analysis/volumetric measurements	Higher PM_2.5_ was associated with smaller WM volumes (in the total brain, association areas, and CC) even in full adjusted models.
			Spatiotemporal model to estimate cumulative PM2.5 exposure (based on air monitoring data, and on participants’ residential addresses) over the 6–7 years preceding MRI.	Standard T1, T2 weighted and FLAIR		
	≥ 65 years					
	WHIM Study				TBV, ventricle, GM, and WM volumes (total and in the 4 lobes), CC, HV, and BG volumes	
						GM, ventricular sizes, HV, and BG did not differ by PM2.5 exposures.
						*Adjustment for ICV, geographical region, age, race/ethnicity, education, income, employment status, alcohol use, smoking, depressive symptoms, BMI, and CVD risk factors.*
[27]	*n* = 934	C	PM _2.5_	MRI 1 T	Lesions assessment	Higher PM2.5 was associated with smaller TCBV and higher odds of CBI.
	≥ 60 years		Spatiotemporal model to estimate average PM2.5 levels (participants’ residential addresses) over 1 year.	T2 weighted double spin-echo coronal sequences		
					WMH and CBI	No clear pattern of association between PM_2.5_ and HV, WMH.
	Framingham Offspring Study				ROI-based analysis/volumetric measurements	
						Associations were no longer significant when adjusted for vascular risk factors (homocysteine, sBP, diabetes mellitus, CVD, history of atrial fibrillation, HT medications, and obesity), but for CBI.
					HV and TCBV	
						*Adjustment for age, sex, time from examination to MRI, income, date of MRI, smoking status, education, and drinking categories* ***.***
			Near roadway exposure			
[16]	*n* = 1365 women	P	PM _2.5_	MRI 1.5 T	Whole-brain voxel-based analysis	Increased PM_2.5_ exposure were associated with smaller volumes in both cortical GM (the bilateral superior, middle, and medial frontal gyri) and subcortical WM areas (the largest clusters were in the frontal lobe, with smaller clusters in the temporal, parietal, and occipital lobes).
			Spatiotemporal model to estimate cumulative PM_2.5_ exposure (based on and air monitoring data, and on participants’ residential addresses) over the 3 years preceding MRI.	Standard T1, T2 weighted and FLAIR		
	71–89 years				GM and WM volumes	
	WHIM Study					
						No statistically significant associations between PM_2.5_ exposure and HV or CC.
						Larger volumes (thalamus, putamen, globus pallidus, and the posterior insula) were associated with increased PM_2.5_ exposures
						*Adjustment for ICV, age, race, BMI, geographic region, education, family income, employment status, smoking, alcohol consumption, CVD history, hypertension, treated diabetes, and prior hormone therapy use.*
[28]	*n* = 236 (with CDR 0–3)	C	PM _2.5_	MRI 1.5 T/3.0 T	Lesions assessment	No pattern of association between residential proximity to major roads or average PM_2.5_ and greater burden of small vessel disease or neurodegeneration.
			Spatiotemporal model to estimate average PM_2.5_ levels (participants’ residential addresses) over 1 year.			
					WMH, cerebral microbleeds	
				T1, T2 weighted, and FLAIR		
					ROI-based analysis/volumetric measurements	
						*Adjusted for age, sex, education, income, smoking status, diagnosis (dementia or other), diabetes, statin, hypertension, and stroke history.*
	74 (12) years					
	MADRC				BPF	
			Near roadway exposure			
*In rats*						
[29]	*n* = 60	L	PM (collected in Lahore, Pakistan).	MRI 4.7 T	Visual inspection	No changes in the hyperintense signal nor any cortical atrophy of brain hemisphere detectable with MRI (even in the group treated with high PM concentrations that showed obvious selective neuronal loss in the cortical areas and glial activations histopathologically).
	4.5 months			T2 weighted RARE sequence		
	Experimental study		Rats were provided with drinking water containing different concentrations of PM.			
			3–4 months of exposure before histopathological assessment and MRI			
				Histopathological assessment: ROIs-based (39 cytoarchitectonally distinct cortical ROIs, 4 caudate/putamen ROIs, and 1 thalamus ROI)		

*Abbreviations*: *BAP* benzo[a]pyrene, *BG* basal ganglia, *BMU* body mass index, *BPF* brain parenchymal fraction, *BREATHE* Brain Development and Air Pollution Ultrafine Particles in School Children, *C* cross-sectional, *CBI* covert brain infarct, *CC* corpus callosum, *CDR* Clinical Dementia Rating, *CVD* cardiovascular disease, *DFC* dorso frontal cortex, *DMN* Default Mode Network, *DTI* Diffusion Tensor imaging, *FA* fractional anisotropy, *FLAIR* fluid-attenuated inversion recovery, *EC* elemental carbon, *FU* follow-up, *GM* gray matter, *ICV* intracranial volume, *HV* hippocampal volume, *HT* hypertension, *L* longitudinal, *MADRC* Massachusetts Alzheimer’s Disease Research Center, *MFC* medial frontal cortex, *MRI* magnetic resonance imaging, *MRS* magnetic resonance spectroscopy, *NO*_*2*_ nitrogen dioxide, *NYC* New York City, *P* prospective, *PAHs* polycyclic aromatic hydrocarbons, *PCC* posterior cingulate cortex, *PM* particulate matter, *ROI* region of interest, *sBP* systolic blood pressure, *SMA* supplementary motor area, *T* tesla, *TBV* total brain volume, *TCBV* total cerebral brain volume (ratio of brain parenchymal volume/total cranial volume), *WM* white matter, *WMH* white matter hyperintensities, *WHIM* Women’s Health Initiative Memory

Four studies were conducted on elderly people in the USA. Some subjects were from the general population [16, 17, 27] and others were elders with concerns about memory loss [28]. These studies presented more homogeneity than those carried out in children in terms of exposure, whether it be for the pollutant, the evaluation method, or the exposure time windows. Indeed, in all studies, the residential exposure to fine particulate matter (PM_2.5_) was calculated using spatiotemporal modeled estimates over 1 to 7 years preceding the MRI assessment. Moreover, the levels of PM_2.5_ were very comparable with a median ranging from 11.0 to 12.2 μg/m^3^.

The neuroimaging outcomes investigated varied widely between the different epidemiological studies. Presence of cerebral lesions considered as markers of small vessel disease [30] (white matter hyperintensities, covert brain infarct, or microbleeds) were examined in a few studies [24, 27, 28]. For the brain’s structures examination, a region of interest (ROI) approach was used in five studies, which focused on measuring defined anatomical structure volumes: gray matter (GM) and white matter (WM) in the whole brain and in the four brain lobes, ventricles, hippocampus, corpus callosum (CC), basal ganglia (BG), and amygdala volumes [17, 18, 25, 27, 28]. Other studies investigated the whole brain and measured at a voxel level (voxel-based morphometry (VBM)), the GM and WM volumes [16, 20, 26], and surfaces [19] using T1-weighted images or the fractional anisotropy (FA) using DTI [20, 26]. Brain functioning, i.e., the neuronal activity measured with fMRI, was only examined in the BREATHE project [20, 26]. Overall, the main confounding factors (i.e., age, sex, and socioeconomic status) were taken into account in the studies’ analyses linking air pollution and brain changes. Analyses aiming at determining the relationships between the observed air pollution-related brain changes and cognition were present only in studies involving children [18–20, 26].

Our review also included a study conducted in rats experimentally exposed to PM collected in Lahore, Pakistan [29]. This study represents the advantage to combine and compare the in vivo MRI findings detected with a visual inspection with the cerebral air pollution-related anatomopathological lesions observed directly on brain sections after animals were sacrificed.

### Air Pollution-Related Brain Changes Detected in Epidemiological Studies

#### Small Vessel Disease

Results from studies interested in MRI lesions reflecting small vessel disease were inconsistent. In the small sample of Mexican children, the frequency of prefrontal white matter hyperintensities was significantly higher in children from Mexico City (56.5%, *n* = 23) than in children living in a less polluted area (7.6%, *n* = 13) [24]. However, there was no clear pattern of associations between PM_2.5_ exposure or proximity of a major road and white matter hyperintensities load in large samples of elderly from the general population [27] or among those with concerns about memory loss [28]. Reflecting, among others, the degenerative changes in small vessels [31], white matter hyperintensities are common findings on MRI in the elderly. Hence, one would have thought to observe a stronger association between TRAP exposure and the white matter hyperintensities burden in older people already subject to the weakening effects of age than in children. These unexpected findings might be due in fact (i) to the measurement methods used to evaluate white matter hyperintensities which are very different in the children studies and in the elderly studies (visual scale [24, 25] versus semi-automated procedure [27, 28]) (ii) and/or to the highly more contrasted levels of TRAP observed in the children studies (Mexico city versus a rural area) than in the elderly studies that showed more limited range of exposure levels.

Nevertheless, significantly higher odds ratios of covert brain infarcts were observed associated with increasing levels of PM_2.5_ [27].

#### Gray Matter

Both studies in children and elders investigating regional brain volumes did not reveal any significant association between air pollution exposure and changes in subcortical brain structure volumes (i.e., hippocampus, amygdala, and basal ganglia) [17, 25]. Results were more inconsistent when studies focused on GM. In rats, while a positive-dose relationship was observed postmortem between PM exposure and severity of neuronal loss on representative brain sections (predominantly in motor cortex and primary somatosensory cortex), no changes in the hyper intense signals nor any cortical atrophy were visually detectable on T2-weighted images [29]. In the birth cohort study conducted in New York City, prenatal exposure to PAHS did not correlate with cortical thickness anywhere across the cerebrum in children [19].

For the elderly participating in the WHIM cohort, whole-brain VBM analysis revealed a link between PM_2.5_ exposure and smaller GM volumes primarily in the dorsolateral and medial prefrontal cortex [16], whereas a prior investigation with a region-based approach failed to find such an association in the same population [17].

#### White Matter

In contrast, a consistent pattern of air pollution-related reduction in WM volumes was observed [16, 17, 19, 25]. Greater PM_2.5_ exposure was associated with smaller volumes in the WM of all association areas (frontal, parietal, and temporal) and the CC of the elderly in the WHIM cohort [16, 17]. In children, there were also significant differences in WM volumes between controls and the Mexico City children in the right parietal and bilateral temporal areas [25]. The authors pointed out that the cognitive deficits in attention, short-term memory, and learning abilities observed in children from Mexico, when compared to the controls, match the localization of the volumetric differences. Neuroimaging investigation of 40 children from New York City revealed a dose-response relationship between increased prenatal PAHs exposure and substantial reductions of the cerebral white matter mostly confined to the left hemisphere of the brain and involving almost its entire surface [19]. Moreover, these WM reductions mediated the deleterious effects of prenatal PAHs exposure on processing speed performance observed in the cohort. Interestingly, distinct effects of PAHs exposure measured later in childhood were detected bilaterally in dorsal prefrontal WM, independently of prenatal PAH exposure.

As a probable consequence of these air pollution-related WM volumes reduction [17], smaller total brain volumes were also observed in the elderly exposed to high levels of PM_2.5_ [17, 27].

#### The BREATHE Study

Among all of the selected studies, the BREATHE project had the advantage of performing different MRI modalities within the same population of primary schoolchildren. Within the 263 children of the study, no significant associations were identified between the measurements of EC and NO_2_ in the school environment and any anatomical, structural, or metabolic brain measurements [26]. However, functional MRI revealed that higher combined levels of EC and NO_2_ were associated with lower functional integration and segregation in key brain networks relevant to both inner mental processes (the default mode network) and stimulus-driven mental operations. Age showed the opposite associations to those of pollution, thus indicating that higher exposure is associated with slower brain maturation. However, within the same group of children, the caudate nucleus appeared to be sensitive to the effects of airborne copper and PAHs exposure. Indeed, airborne copper was associated with tissue modifications in the caudate nucleus, which presented higher GM concentrations probably at the detriment of WM [20]. In addition, potentially detrimental effects of copper on the WM pathways in the caudate nucleus region and on functional connectivity between the caudate nucleus and the frontal lobe operculum were revealed by DTI and fMRI analyses, respectively. In addition, exposure to PAHs, and in particular to benzo[a]pyrene, was associated with a subclinical reduction in the caudate nucleus volume [18].

## Discussion

The studies integrating neuroimaging and epidemiology revealed that long-term exposure to air pollution might have adverse impacts on brain structures and functioning in the general population and that these impacts are detectable with different MRI modalities. Moreover, findings from all these studies suggest that, because of their localization and their magnitude, these air pollution-related brain changes visualized in vivo could mediate the effects of air pollution on cognition.

So far, in vitro and in vivo animal studies have focused primarily on the effect of TRAP on GM and have revealed impaired synaptic functions [10], alterations in neuron morphology [32], and neuron death [13, 29, 33, 34] subsequent to the exposure to the TRAP components. Studies investigating the impact of such an exposure on myelin, the main component of WM, are still scarce [15, 35]. However, throughout the literature reviewed, it appears that WM might be particularly vulnerable to TRAP neurotoxicity. High levels of airborne PM_2.5_, PAHs or living in a highly polluted city were associated with reductions of WM, especially the WM of the frontal lobe [16, 17, 19, 25]. These associations were detected in both children and elderly using different MRI methods and with exposure assessed during different time windows (i.e., prenatal for PAHs, old age for PM_2.5_, and life span for children living in Mexico City). In children and elderly, the oligodendrocytes (cells that produce the myelin sheath in the CNS) are sensitive to inflammation and oxidative stress [36, 37]. Both processes are thought to be key component of TRAP neurotoxicity [38]. However, the reasons explaining their vulnerability are certainly different depending on the person’s stage of life. In children, during the prenatal period, the pre-oligodendroglial cells first increase and settle along the axons. After maturation, the oligodendrocytes start the real myelination process which occurs in a very precise spatiotemporal way in the brain [39]. This process starts at the third trimester of pregnancy. Myelination remains intense during the first 2 years of life and is protracted, to a lesser extent, until adulthood [40, 41]. Because of these dramatic changes concentrated over the fetal period and the very first years of life, prenatal exposure is likely to have a more severe impact on WM, as suggested in the birth cohort conducted in New York City [19] and also in mice presenting hypomyelination and aberrant WM structural integrity after early-life exposure to ultrafine particle (UFP), during a period equivalent to the human 3rd trimester of pregnancy [35]. No WM change was observed in the primary schoolchildren exposed to high levels of TRAP in BREATHE [26], though in this cohort, both WM volumes and WM tract architecture were investigated using morphometric approaches and DTI, respectively. It is possible that the BREATHE study, in measuring air pollution in late childhood, did not capture the sensitive exposure time windows to explore the effects of TRAP on WM. In contrast, at the other end of the age spectrum, WM volume decreases rapidly from 60 years onwards [42]. Aging makes the oligodendrocytes more prone to degeneration, resulting in increased myelin breakdown [43]. On the other hand, age-related vascular changes lead to decreased blood flow [44, 45] and hence increase WM intrinsic vulnerability to hypoperfusion [46]. In this context, and considering that exposure to PM_2.5_ is likely to cause alterations in cerebrovascular hemodynamics [47], TRAP may be suspected to have detrimental ischemic effects on WM in the elderly. However, the absence of correlations between white matter hyperintensities due to ischemia [31, 48] and exposure to PM_2.5_ observed so far in two different populations of old people [27, 28] does not support this biologically plausible hypothesis.

Regarding GM, cortical GM already shows widespread thinning under the effect of aging in the elderly [49] and appears to be susceptible to the additional harmful effects of TRAP [16, 17]. In contrast, no TRAP effect on the children’s cortical GM was detected [19, 25, 26]. The dynamics of the cortical GM volume is complex in children as GM volume increases from early childhood to later childhood (6–9 years) and decreases thereafter [50]. Therefore, cross-sectional imaging may not be appropriate to detect, or even interpret, any TRAP-related GM volume changes in children, whatever is the considered exposure time window [19, 25, 26]. The exploration of GM volume trajectory in longitudinal studies could be much more relevant with children.

Among subcortical structures, basal ganglia was the only component to present tissue alterations and size reduction in relation to traffic-related pollutants: specifically airborne copper and PAHs exposure in children [18, 20]. These observations made in an urban context echo findings from occupational studies suggesting that basal ganglia might be a specific target of inhaled transition metals [51–58]. Indeed, occupational studies using neuroimaging have highlighted that several metals including copper, manganese, and iron present in great amounts in welding and smelting fumes, tend to preferentially accumulate in the basal ganglia of workers [51, 53–57], and could have a potential local neurotoxicity [55–57]. Furthermore, in this brain region and more particularly in the striatum (caudate nucleus and putamen), the neuronal activity is mainly modulated by dopamine, a neurotransmitter prone to oxidation and with a metabolism that serves as a major source of intracellular reactive oxygen species [59]. Therefore, the overstretched local antioxidant systems in the striatum [60] are likely to have difficulties dealing with any additional oxidative challenge like exposure to PAHs [9, 33], and this depletion possibly makes this region particularly vulnerable to TRAP [9, 61].

The structural changes described above are more likely to appear after long-term exposure. In contrast, current or short-term exposure may influence the fluctuating brain activity, as suggested by synaptic dysfunction observed in mouse hippocampus after a 2-h exposure to PM [10], as well as by the changes reported in human volunteers’ electroencephalograms following a 1-h exposure to diesel exhaust [62]. Furthermore, when anatomical MRI did not reveal any associations between levels of exposure in the school environment and the children’s brain structures in the BREATHE study, fMRI was sensitive enough to detect a delay in the brain’s functional organization related to air pollution exposure [26]. FMRI may prove to be a useful tool to investigate the effects of short-term or current exposure to air pollution on brain and thus could help to better understand the relationship between acute exposure and disorders in attention processes [63] and in visual information processing speed [64] observed in epidemiological studies.

In preclinical Alzheimer’s disease, connectivity disorders are observed years before brain anatomical changes [65]. It is yet to precisely identify what are the impacts of TRAP on the neuronal activity in children, as well as to determine whether these TRAP-related brain functions impairment might lead later to detectable structural modifications. This is an important question to address because if this is the case, it would emphasize the necessity to study both the pre- and the postnatal exposure periods when assessing the effects of TRAP on neurodevelopment.

The cognitive correlates of these air pollution-related brain changes were observed in children. Pujol et al. reported that the performance in motor speed and in motor response consistency showed correspondence with the identified functional networks and the WM tracts affected by TRAP [20, 26]. Peterson et al. [19] showed that the magnitude of the reductions in WM surface mediated the association between decreased performance in processing speed and PAHs exposure. Interestingly, the authors also reported that these neuroimaging findings were associated with conduct disorders and ADHD symptoms. In the restricted MRI subsample (*n* = 40), the association between ADHD symptoms and PAHs exposure was not significant and mediation analyses were not possible. However, considering that in the whole sample (*n* = 250) this association was significant, a potential mediator effect of WM reductions between PAHs exposure and ADHD symptoms could be assumed.

These investigations were obviously limited by the batteries of cognitive tests designed for the cohorts. But considering that the neuroimaging findings mostly concern WM and the prefrontal cortex [16, 17, 24, 25], the possibility of subsequent cognitive impairment is extended to many domains including all of those reported in epidemiological studies [2•, 5]. The prefrontal cortex is associated with higher cognitive functions such as working memory, episodic memory retrieval, and executive function [66]. On the other hand, WM interconnects widely distributed networks that might be involved in all cognitive domains [67].

### Recommendations for Future Studies

**Fig. 2:**
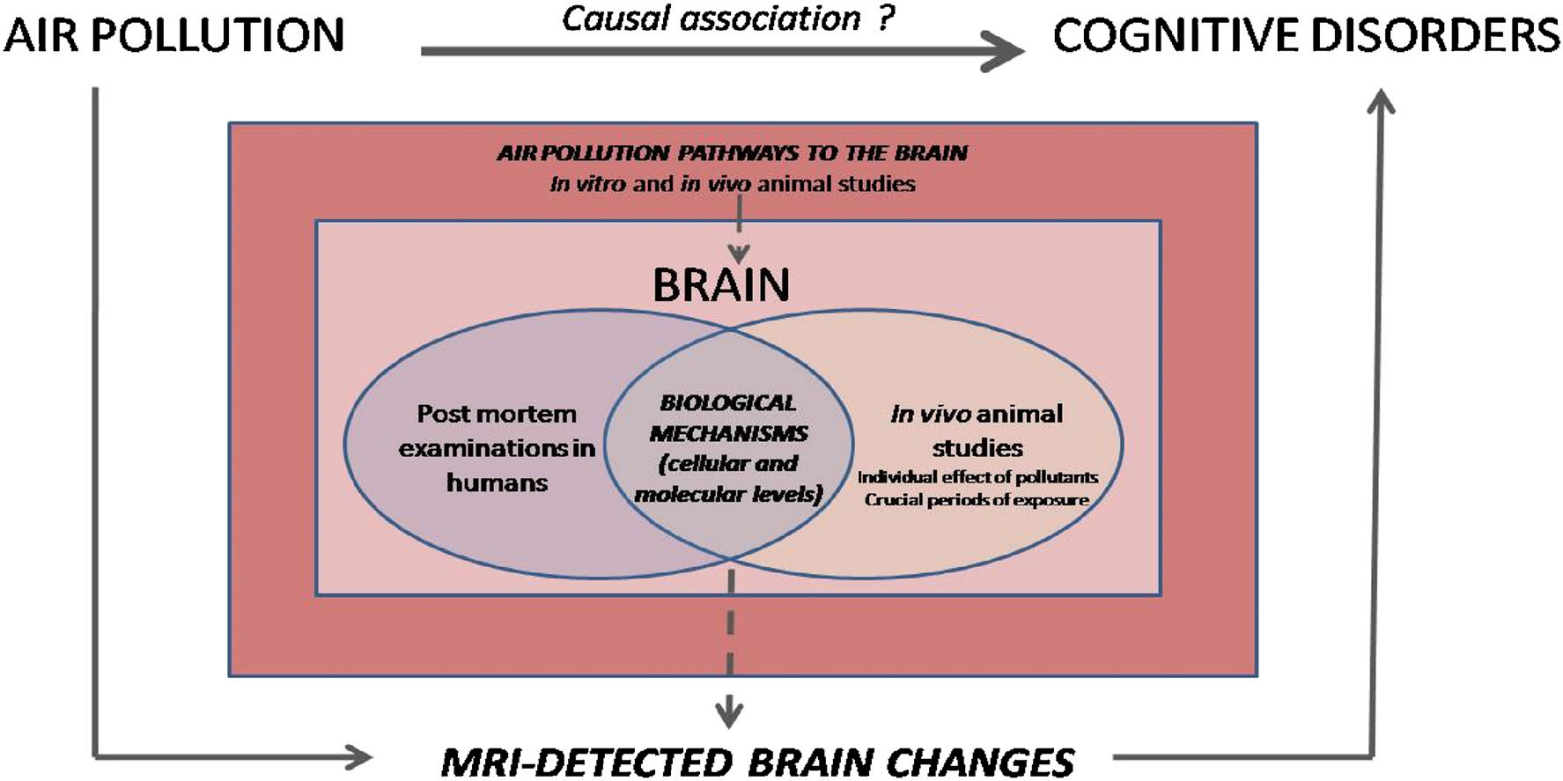
Understanding the link between air pollution and cognition: the central role of studies interfacing environmental epidemiology and neuroimaging in humans

Despite a great heterogeneity in study populations, MRI methods, pollutants, and cognitive investigation, the studies reviewed suggest a biological plausibility in the associations between exposure to urban air pollution and cognition observed in the overall population [2•, 5, 6]. From only a handful of studies, insights drawn are already promising and should encourage a more systematic use of neuroimaging in epidemiological studies interested in the question of the effects of air pollution on the human brain (Fig. 2). As it is still an emerging field, areas of improvement exist for future studies. The first aspect to take into account should be the optimization of the MRI detection sensitivity. The brain has a dynamic structure in constant evolution throughout life [50], so longitudinal studies with repeated MRI sessions should be promoted in order to assess the impact of air pollution on the developmental/aging trajectories of WM and cortical GM. Subcortical structures should be examined with methods that are able to better capture the complexity of their anatomy, often made up of several subfields, each with a partially independent functional role [68]. Recent methods permit the exploration of the brain structure’s shape and beyond purely volumetric measures [69]. These structures merit a particular interest because of their involvement in many cognitive processes and psychiatric disorders [70–72].There is growing evidence suggesting that the fetal period is a highly vulnerable period [19, 35]. However in humans, MRI data investigating the brain damages consecutive to this period have only been collected during childhood so far [19]. MRI sessions earlier in life (i.e., prenatal or early postnatal MRI [73]) should be encouraged to get better insights about the effects of prenatal exposure on the brain. Regarding cognition, mediation analyses should be performed whenever this is feasible in order to determine the exact influence of the brain damages.

Yet, there are remaining questions that still require experimental and neuropathological investigation before any conclusions can be made about a causal link between urban air pollution and cognitive disorders in humans. Tremendous work has been done and is still ongoing to determine the mechanisms through which air pollutants reach the brain [14, 74] and induce damages at the cellular and molecular level [11••, 74]. Current findings converge towards the hypothesis that TRAP neurotoxicity leads to neuroinflammation, oxidative stress, and/or neurovascular unit dysfunction, which in turn result in oligodendrocytes and/or myelin damage and loss of neurons or alterations in their morphology [29, 32, 34, 35, 75]. Currently, we can only assume that MRI-detected changes reflect these cellular damages. To date, only Ejaz et al. examined whether imaging-pathologic correlations might exist in rats and failed to find any brain changes by visual inspection of the MRI images while pathological analyses revealed neuronal loss [29]. The investigation of this type of correlation with more advanced MRI methods in animal studies is necessary to better understand the biological meaning of the MRI-detected brain changes in humans (Fig. 2). Besides, because humans are exposed throughout life to a mixture of air pollutants, in vitro and in vivo animal studies are the only ones that have the possibility to determine the individual effect of the different chemical components of air pollution, as well as the key exposure time window.

To summarize, future studies integrating neuroimaging and epidemiological studies should consider (i) to explore the effects of TRAP on brain growth/aging trajectories using repeated MRI sessions, (ii) to perform MRI early in life, (iii) to investigate the associations between data from fMRI (task and at rest) and acute exposure to TRAP estimated in 2–3 days before the fMRI session, and (iv) to explore all the potential neuropsychological functioning (psychomotor, cognition and social-emotional) [76] correlates of the TRAP-induced brain changes.

## Conclusion

In conclusion, studies integrating environmental epidemiology and neuroimaging suggest that WM, cortical GM, and basal ganglia could be targets of TRAP and that these detected TRAP-induced brain damages might underlie the observed association between TRAP exposure and cognitive disorders in humans. Related to several fields of research, these studies still face many methodological challenges. They remain, however, the only means to investigate in vivo the physical effects of TRAP potentially involved in cognitive, behavior, and psychomotor disorders in humans. Fortunately, areas of improvements have been identified and are feasible. Future studies, together with inputs from experimental findings, should provide more relevant and detailed knowledge about the nature of the link between TRAP exposure and cognitive, behavior, and psychomotor disorders observed in the general population.
